# Establishing microbial communities to promote the growth of *Pleurotus ostreatus* through a top-down approach is hindered by the dominance of antagonistic interactions

**DOI:** 10.1128/aem.00898-25

**Published:** 2025-08-25

**Authors:** Marie Bonduelle, Frédérique Desrochers-Noiseux, Audrey-Anne Durand, Simon Barnabé, Philippe Constant

**Affiliations:** 1 Institut national de la recherche scientifique, Centre Armand-Frappier Santé Biotechnologie 14851, Laval, Québec, Canada; 2 Université du Québec à Trois-Rivières, Trois-Rivières, Québec, Canada; Royal Botanic Gardens, Surrey, United Kingdom

**Keywords:** top-down assembly of microbial consortia, *Pleurotus ostreatus*, microbial enrichment, antagonism, lignocellulose

## Abstract

Few attempts have been made to examine the diversity and functions that characterize microbial communities compatible with and beneficial to the biotechnologically relevant mushroom *Pleurotus ostreatus*. The quest for complementarity is complicated by the variable nature of beneficial traits, impairing the rational assembly of synthetic communities to improve bioprocesses. This study investigated whether the compatibility between *P. ostreatus* and microbial enrichments is lessened in conditions favoring saprophyte metabolism and is enhanced when a combination of recalcitrant and labile carbon is integrated in the enrichment. The microbial diversity of enrichment cultures and substrates colonized by *P. ostreatus* was analyzed through PCR amplicon sequencing, and the proliferation of *P. ostreatus* was assessed by quantitative image analysis. In general, co-inoculation of lignocellulosic substrate with microbial enrichment reduced the growth of *P. ostreatus*. The saprophytic enrichment conditions were more conducive to the development of antagonistic communities inhibiting *P. ostreatus* growth than enrichment cultures integrating labile carbon substrates. Both microbial community analyses and *in vitro* assays led to the identification of a single phylotype affiliated with *Brevundimonas* spp., which displayed neutral interaction with *P. ostreatus*. Recalcitrant and labile carbon degradation functions were not primary factors driving beneficial microbial communities for *P. ostreatus*. Additional functions beyond carbon metabolism are likely to promote beneficial interactions. Directed enrichment cultures, integrating the mushroom in the earliest stage of the procedure, are expected to promote more beneficial interactions than top-down approaches.

**IMPORTANCE:**

Lignocellulosic biomass upcycling biotechnologies integrating solid-state fermentation by fungi are aligned with sustainable development perspectives. While the recalcitrance of this biomass imposes a challenge for the implementation of these bioprocesses converting the lignocellulosic feedstock into bioenergy and bioproducts, pretreatment of lignocellulose biomass with fungi is efficient and generates fewer by-products than chemical approaches. Optimization and stabilization of this bioprocess by integrating microbial consortia has received little attention. The significance of our research is to bridge that knowledge gap by examining how interactions between the biotechnologically relevant basidiomycete *Pleurotus ostreatus* and microbial communities influence fungal growth in lignocellulosic substrate. Directed enrichment cultures integrating *Pleurotus ostreatus* as a selective agent are expected to trigger more beneficial interactions promoting mushroom growth than our top-down approaches, due to a dominance of antagonistic mushroom-bacteria interactions.

## INTRODUCTION

Interactions between bacteria and fungi (BFI) underpin major ecological processes, essential for ecosystem functioning, including the health of host organisms (animals and plants) and biogeochemical cycles. Thus, BFI are increasingly studied in medicine, agriculture, environmental science, food processing, and biotechnology (1, 2). Antagonistic relationships are particularly represented, with many well-characterized interactions ranging from competition for nutrients to interference competition (3). Despite the dominance of negative inter-kingdom interactions, positive interactions such as mutualism, commensalism, or symbiosis contribute to plant growth promotion through arbuscular mycorrhizal fungi (4, 5) and organic waste upcycling biotechnologies integrating solid-state fermentation (6). The latter is particularly aligned to sustainable development perspectives, integrating the valorization of lignocellulosic biomass generated by agro-industrial activities.

Due to its high carbon density and availability, lignocellulosic biomass could reduce dependency on non-renewable resources. The recalcitrance of the substrate imposes a challenge for the implementation of bioprocesses converting the lignocellulosic feedstock into bioenergy and bioproducts (7). Unlike chemical methods, pretreatment of lignocellulosic biomass using fungi that produce cellulase, hemicellulase, and lignin-modifying enzymes is more efficient and generates fewer by-products (8). However, this bioprocess is less commonly applied in industry, as microbial growth slows down the production rate. Other drawbacks relate to the energy costs associated with biomass sterilization requirements (9). Nevertheless, the demand for more environmentally friendly industrial processes, combined with the global challenge of biomass management, positions biological pretreatment as a promising solution for the future. Optimizing bioprocess performance through the characterization of microbial communities that are compatible with and beneficial to fungal growth is therefore essential to control the residual microbial community in the biomass after cost-effective pasteurization and to shorten the fungal growth period, respectively.

Lignocellulosic biomass is a highly resistant substrate when it comes to degradation because of its chemical composition, with lignin displaying a complex and irregular structure, and due to the crystallinity of cellulose (10). Despite this natural recalcitrance, bacteria and fungi developed several strategies supporting the carbon cycle, such as the production of extracellular enzymes and the deployment of oxidative processes for nutrient acquisition and for lignin and polysaccharide recycling (11). *Pleurotus ostreatus* is a saprophytic fungus with a broad ecological niche, with species from this complex found in both grassland and forest ecosystems, extracting nutrients from grasses and decaying wood (12, 13). Beyond the degradation of cellulose and hemicellulose, *P. ostreatus* and white rot fungi in general have the unique ability to mineralize lignin to CO_2_ and water (14). Bacterial communities surrounding *P. ostreatus* also demonstrated cellulose and hemicellulose degradation capabilities (15, 16). Laccase-like genes were identified in *Pleurotus* sp. associated bacteria, suggesting that bacteria are also involved, at least in part, in lignin mineralization (17). This functional redundancy in the degradation of organic compounds has been observed not only in lignocellulosic biomass but also in other ecosystems such as litter (18) and soil (19). Consequently, there is competition between bacteria and fungi for the occupation of the ecological niche. Competition for carbon is stronger than for other resources due to the overlap between bacterial and fungal demand for organic compounds (19). While bacteria are more efficient for labile organic compounds assimilation, fungi outcompete the former for complex compounds thanks to their high catalytic enzyme efficiency (19). The extensive mycelial network of fungi allows nutrient bioprospection stored in recalcitrant organic pools (20), while the release of sugar monomers by fungal extracellular enzymes leads to inter-kingdom competition, the dominant soil interaction (19). This competition appears to be even more pronounced in an environment with limited resources, such as lignocellulosic biomass (21, 22).

Despite this competition, bacteria and fungi co-exist in ecosystems, and their metabolic dependency and complementarity shape their co-occurrence in nature (23). One mechanism is cross-feeding with lignocellulose-degrading synthetic communities (24, 25), for which bacteria and fungi can both benefit from the metabolites released by the partner. These observations drive studies for bacterial inoculation of mushroom growing substrate to enhance their biomass yield (26). Secondary metabolism functions and traits exerting a growth-promoting effect were examined in the literature (15, 26–30). The ability to fix nitrogen (31), or to produce 1-aminocyclopropane-1-carboxylic acid deaminase (32) and indole-3-acetic acid (33), is an example of beneficial bacterial traits for fungal growth. The ability to produce volatile organic compounds (34) and siderophores (35) was also reported to be involved in *Pleurotus* sp. growth promotion. The exact mechanisms underlying bacteria and fungi interactions, however, are largely uncertain because they are context-dependent (36) and sometimes conflicting (29). The identified traits are primarily based on correlations, without direct functional evidence, indicating that most mechanisms remain unproven.

Predicting beneficial interactions for *P. ostreatus* growth and defining an efficient enrichment strategy tailored to beneficial functions in bioprocesses is challenging. Isolating beneficial communities for *P. ostreatus* from natural environments is complicated by the unique nature of agricultural lignocellulosic waste integrated in biorefinery, i.e. microbial assembly in decaying wood or spent mushroom substrate (36) differs from those in coco fiber in a greenhouse containing plant tissues, fertilizers, and chemicals. In addition, this design is impeded by the poor understanding of the delicate trade-off delineating bacterial beneficial complementarity supporting vegetative growth of the fungus and competition reducing fungal biomass. Finally, bacterial communities surrounding fungi have been studied using high-throughput sequencing and imagery technologies (23, 37), but few attempts have been made to examine the diversity and functions that characterize microbial communities compatible with and beneficial to fungal growth. This study bridges that knowledge gap by examining how interactions between the biotechnologically relevant basidiomycete *Pleurotus ostreatus* and microbial communities influence fungal growth in coco fiber from planting substrates of a commercial greenhouse.

The absence of selective traits beneficial to the fungus impedes a targeted enrichment strategy. However, competition interactions for access to carbon and energy sources under saprophytic conditions are expected. Under this hypothesis, a microbial enrichment relying on lignocellulosic biomass as a sole carbon source is likely to be less compatible with *P. ostreatus* than enrichment strategies including additional labile carbon sources. In the latter, the enrichment of microbial consortia compatible with fungus growth would support beneficial traits. To test this hypothesis, contrasting microbial consortia were assembled through a top-down approach. Six microbial enrichments were first obtained under elevated or ambient CO_2_ conditions and with different carbon amendments, ranging from recalcitrant only to a mixture of recalcitrant and labile carbon sources. *P. ostreatus* was then inoculated alone or with microbial enrichment cultures in the coco fiber retrieved from a commercial greenhouse. The microbial diversity in the enrichment cultures and in the substrate with *P. ostreatus* was analyzed to relate fungal growth performance with the composition and diversity of microbial communities.

## MATERIALS AND METHODS

### Sample collection and composite inoculum preparation

Soil, compost, and wood chips were sampled and aggregated into a composite inoculum for enrichment cultures. A soil mixture was first prepared with 100 g surface soil samples collected in a public park located in Montreal (Canada). Surface soil samples and decomposing wood chips comprising visible mycelium on the surface were collected in a maple forest located in Laval (Canada). Three samples of composted bean plant residues were supplied by a collaborator (Solinov Inc., Brossard, Canada). The different samples (soil, compost, and wood chips) were mixed in a ratio of 1:1:1 and used as inoculum in the enrichment cultures.

Coconut fiber retrieved from pepper production wastes (Les Fermes Luffa, Montréal, Canada), used as residual lignocellulosic biomass in this study, was also included as inoculum for enrichment cultures.

### Enrichment cultures

**Fig 1:**
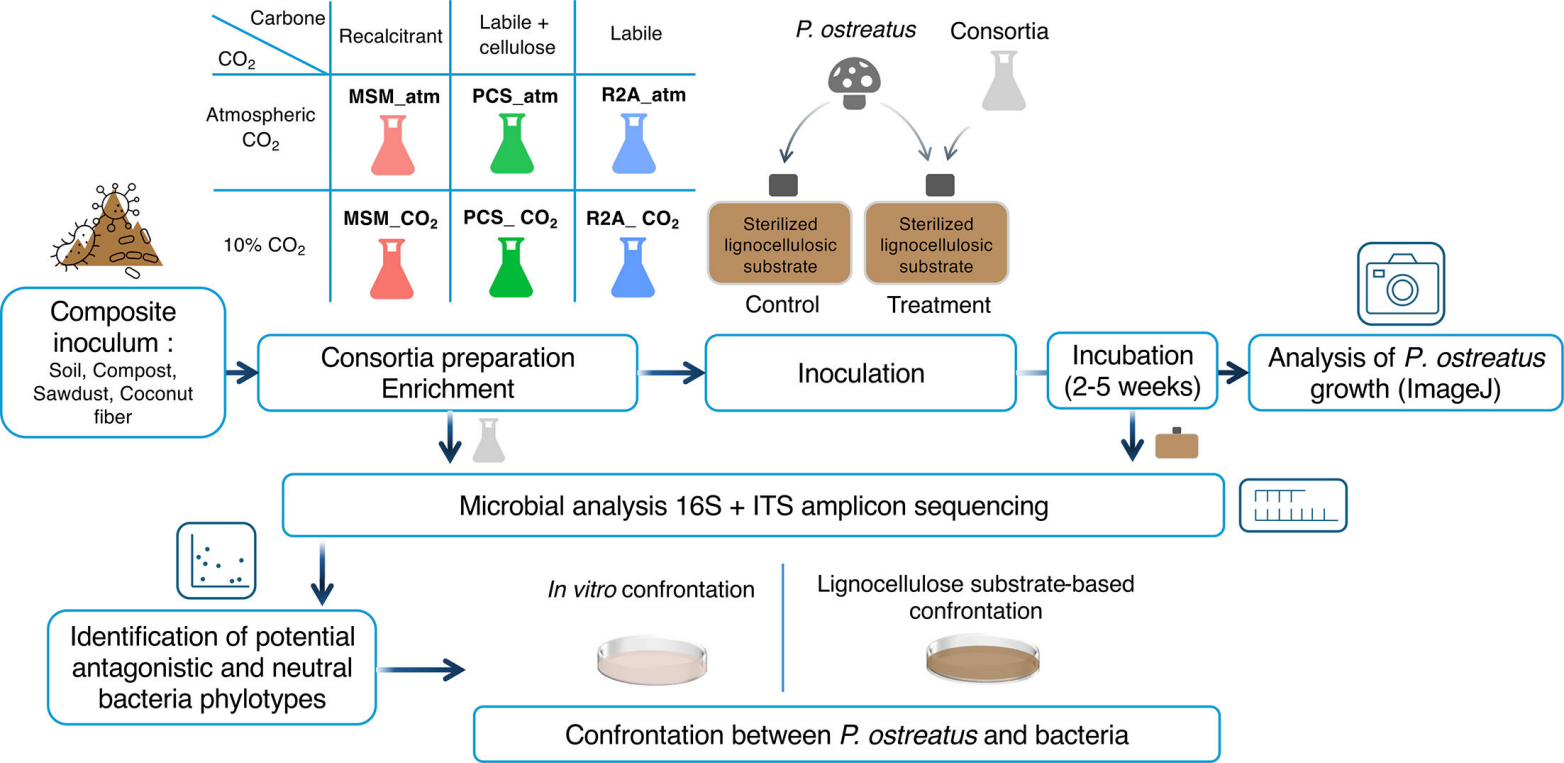
Schematic overview of the experimental design.

Inoculum and non-sterile coconut fiber (1% wt/vol) were transferred into Erlenmeyer flasks containing Reasoner’s 2A medium (R2A), peptone cellulose salt medium (PCS), and minimal salt medium (MSM) (Table S2) (38). The non-sterile coconut fiber was expected to promote the growth of endogenous lignocellulosic-degrading microorganisms and was the only source of carbon for the MSM medium. A sterilized cellulose paper strip (0.3 g) was added to PCS cultures. MSM cultures provided recalcitrant carbon sources, while R2A and PCS offered more labile carbon inputs, with PCS expected to promote the growth of cellulose-degrading microorganisms due to the presence of cellulose paper supplementation. Enrichments were incubated under two different atmospheres: ambient atmosphere through porous foam plugs placed on the apertum of the flasks (MSM_atm, R2A_atm, PCS_atm) or under a static headspace comprising elevated CO_2_ concentration (MSM_CO_2_, R2A_CO_2_, PCS_CO_2_) (Fig. 1). CO_2_ was manipulated to promote complementary functions related to carbon fixation metabolism absent in mushrooms. Concentrations of approximately 10% CO_2_ were achieved by injecting a defined volume of synthetic gas (99.9% CO_2_, Bone Dry 3.0, absolute dry) using a gastight syringe (Precision Analytical Syringe, Pressure-Lok). The average CO_2_ concentration was 9.2%, as measured by GC-FID (Agilent Technologies model 7890B) (39). All enrichment cultures were performed with three independent repetitions. Flasks were incubated at 25°C under agitation at 160 rpm for 10 days (3 media × 2 gaseous conditions × 3 repetitions = 18 enrichment cultures). After 10 days of incubation, the total volume of the microbial cultures was transferred to 250 mL of fresh sterile medium (R2A, MSM, or PCS) with 1% non-sterile coconut fiber and a cellulose filter paper strip in cultures with PCS medium. All flasks were incubated at 25°C under agitation at 160 rpm for 10 days. These enrichment cultures were sampled when the cell density reached at least 5 log cells/mL as measured by the most probable number method. Cellulose filter paper strips, used as an indicator for cellulase activity in PCS cultures, were visually degraded. Two sample fractions were collected from the cultures. The first fraction (1.8 mL) was centrifuged at 10,000 *× g* for 10 min, and the pellets were stored in 2 mL tubes at −20°C for subsequent DNA extraction. The remaining volume was centrifuged at 5,000 *× g* for 10 min at 20°C. The supernatant was discarded, and the pellet was washed twice in phosphate-buffered saline solution (PBS) and resuspended in a final volume of 100 mL 1× PBS solution. These microbial suspensions were utilized to inoculate lignocellulosic substrate.

### Lignocellulosic substrate preparation

The lignocellulosic substrate was prepared by mixing by hand 1,250 g sawdust with 1,250 g coconut fiber, 50 g calcium oxide, 250 g wheat bran, and 7.5 L reverse osmosis-filtered water to obtain a moisture content of 60% and a pH of 6.5. Then, 500 g lignocellulosic substrate was transferred into an autoclavable bag. The bags were closed with a plastic collar and a cap with a membrane to allow sterile air exchange (Fig. S1). A total of 24 bags were then sterilized and left to cool for 12 h before inoculation.

### Inoculation

Fermentation bags were inoculated aseptically by adding 10 g solid fungal inoculum (mycelium of *P. ostreatus* cultivated in sterile rye grains) and 50 mL washed microbial suspension retrieved from the enrichment cultures. Sterile substrate inoculated with *P. ostreatus* without microbial inoculum and sterile substrate without *P. ostreatus* inoculum and without microbial inoculum were included as controls. This completely random design represented a total of 24 bags: (6 consortia + 2 controls) × 3 repetitions. Inoculated bags were randomly placed in an environmental chamber (Growth Cabinet, SANYO) in darkness, at 23°C and 70% humidity.

### Fungal growth measurement

After 2 weeks, control bags inoculated with *P. ostreatus* without microbial inoculum were retrieved and stored at 4°C. The substrate surface of these control bags was completely covered with mycelium. Bags inoculated with *P. ostreatus* and microbial inocula were kept in the chamber for an additional 3 weeks to allow fungal growth. At the end of incubation, compact substrates were removed from their fermentation bags (control and treatment) for photograph capture of their front face and sampling. The substrate surface area covered by the mycelium of *P. ostreatus* was measured using ImageJ software (version 13.0.6) (40). The hue, the brightness, and the saturation were adjusted, and the area of the substrate not covered by mycelium was obtained after converting the threshold color into binary. The difference between the total surface area of the substrate and the surface area not covered by mycelium was used to deduce the substrate surface area covered with mycelium. The latter was converted into a percentage of mycelial growth relative to the substrate surface.

### Sampling

Because of the heterogeneity of the lignocellulosic substrates, a composite sample of colonized substrate (20 g) was assembled by mixing subsamples collected from ten positions within the substrate. Composite samples representative of each experimental treatment were stored at −20°C for subsequent microbial diversity analyses.

### Microbial diversity analysis

Total DNA was extracted from enrichment cultures and composite samples of lignocellulosic substrates with the DNeasy PowerLyzer Microbial kit and the DNeasy Plant Pro Kits (QIAGEN), respectively, following the manufacturer’s instructions. Bacterial and fungal communities were examined by PCR amplicon sequencing. Universal primers 16S_520F (5′ AGCAGCCGCGGTAAT 3′) and 16S_799R2 (5′ CAGGGTATCTAATCCTGTT 3′) (41) were used to target the V4 region of bacterial 16S rRNA gene (279 bp), whereas the primers ITS1F (5′ CTTGGTCATTTAGAGGAAGTAA 3′) and 58A2R (5′ CTGCGTTCTTCATCGAT 3′) (42) were used for the fungal ITS1 region (300 bp). The libraries were prepared as described in reference 43. A single replicate of lignocellulosic substrates inoculated with the R2A_G enrichment was not included in the libraries due to insufficient PCR amplicon concentration. Pooled libraries were subjected to Illumina MiSeq PE-250 sequencing (Genome Québec). The raw sequence reads were processed in the software RStudio (version 4.2.0) (44). Illumina barcodes and primers were removed after trimming using Cutadapt (version 4.6) (45). The sequence data were then processed using the DADA2 package (version 1.26.0) (46). After dereplication, merging paired forward and reverse reads, and removing chimeras, an amplicon sequence variant (ASV) table was constructed. Taxonomy was assigned to ASVs using the reference database Silva version 138.1 for bacterial 16S rRNA genes (47) and the database UNITE version 29.11.2022 for fungal ITS (48). Only ASVs representing at least 0.005% of the whole ASV tables were kept for subsequent analyses (49). A total of 1,051 ASVs were obtained for the 16S rRNA gene, and 195 ASVs were obtained for the ITS (Tables S3 and S4).

### Confrontation between *P. ostreatus* and bacteria *in vitro*

After the analyses of the microbial community in the lignocellulosic substrate, potential antagonistic, neutral, or auxiliary phylotypes on *P. ostreatus* growth were identified. These results were further tested in bioassays. Bacterial isolates affiliated with the same genera were selected to evaluate if potential antagonistic and auxiliary effects on *P. ostreatus* are conserved among different species. *Pseudomonas* sp. MB65 (PQ642331)*, Rhizobium* sp. MB37 (PQ642303), and *Brevundimonas* sp. MB49 (PQ642315) was selected in an in-house collection of bacterial isolates. *Asticcacaulis endophyticus* ZFGT-14 (BCCM 27605) and *Shinella fusca* WF29 (BCCM 24714) were obtained from the Belgian Coordinated Collections of Microorganisms (BCCM) (Tables S5 and S6). A mycelial plug of *P. ostreatus* (75 mm diameter) was collected from the edge of the growing colony after seven incubation days on a PDA agar plate (Millipore) and was placed at the center of R2A agar plates (Millipore). Four drops (10 µL) of overnight bacterial cultures in R2A broth were inoculated at four points placed within a distance of 2.5 cm from the center of the mycelial plug. Each single bacterial strain and control (sterile R2A broth) was represented by three independent repetitions. All plates were incubated at 25°C, and the growth of *P. ostreatus* mycelium was evaluated by measuring the area of the mycelium (mm^2^) with ImageJ. The mycelium surface area was then converted into the relative growth percentage of *P. ostreatus* mycelium co-cultured with each bacterial strain relative to *P. ostreatus* mycelium alone without bacteria. Interactions between *P. ostreatus* and *Brevundimonas* sp. MB49 was further examined in a lignocellulose substrate-based confrontation assay. The assay and the protocol to assess the growth of the bacteria by droplet digital PCR (ddPCR) in the presence and in the absence of *P. ostreatus* are described in the supplemental material (Fig. S2).

### Statistical analysis

Statistical analyses were performed with the software RStudio (version 4.2.0) (44). Analysis of variance (ANOVA) and *post hoc* Tukey tests were performed to compare α-diversity among the different treatments, *P. ostreatus* mycelium in the fermentation bags among the different treatments, *P. ostreatus* mycelium area in the *in vitro* confrontation assay, and *Brevundimonas* sp. MB49 abundance in the lignocellulose substrate-based confrontation assay among the different treatments. The α-diversity was calculated using the sample size and coverage-based rarefaction and extrapolation (R/E) of the Hill numbers of species, richness (q = 0), Shannon index (q = 1), and inverse of Simpson’s concentration index (q = 2) using the package iNEXT (version 3.0) (50). β-diversity analyses were computed on transformed ASV tables with the *decostand* function (Hellinger normalization). Principal coordinate analyses (PCoA) were performed on the Bray-Curtis distance matrix to examine dissimilarity of microbial community composition of the different enrichment cultures and lignocellulosic treatment conditions with the package phyloseq (version 1.42.0). Dissimilarities were further tested with permutational multivariate analysis (PERMANOVA) with the adonis2 function in the package vegan (version 2.6-4) (51). The differential abundance of ASVs between treatments was tested through the analysis of composition of microbiomes with bias correction (ANCOM-BC) with the package ANCOMBC (version 2.0.3) (52). Microbial communities were constrained by the culture medium used in enrichment (MSM, R2A, or PCS), the CO_2_ condition, the relative proportion of ASVs affiliated to *Pleurotus* sp., and the percentage of *P. ostreatus* mycelial growth in a Bray-Curtis distance-based redundancy analysis (db-RDA). The function *forward.sel* from the package *adespatial* (version 0.3.23) was used for parsimonious db-RDA (53).

## RESULTS

### Diversity of the microbial enrichments

The integration of three different nutrient formulations, ranging from recalcitrant to labile carbon sources, with or without elevated CO_2_, was successful in achieving contrasting microbial enrichments. The cultivation media significantly influenced the α-diversity of both bacteria and fungi, with bacteria showing the strongest response (Table S7). The MSM medium was the most favorable condition promoting bacterial species richness and Shannon diversity, while the labile carbon sources integrated in PCS and R2A were favorable for a few dominant ASVs, leading to a reduction of α-diversity. Neither bacterial nor fungal communities responded to CO_2_ treatments.

**Fig 2:**
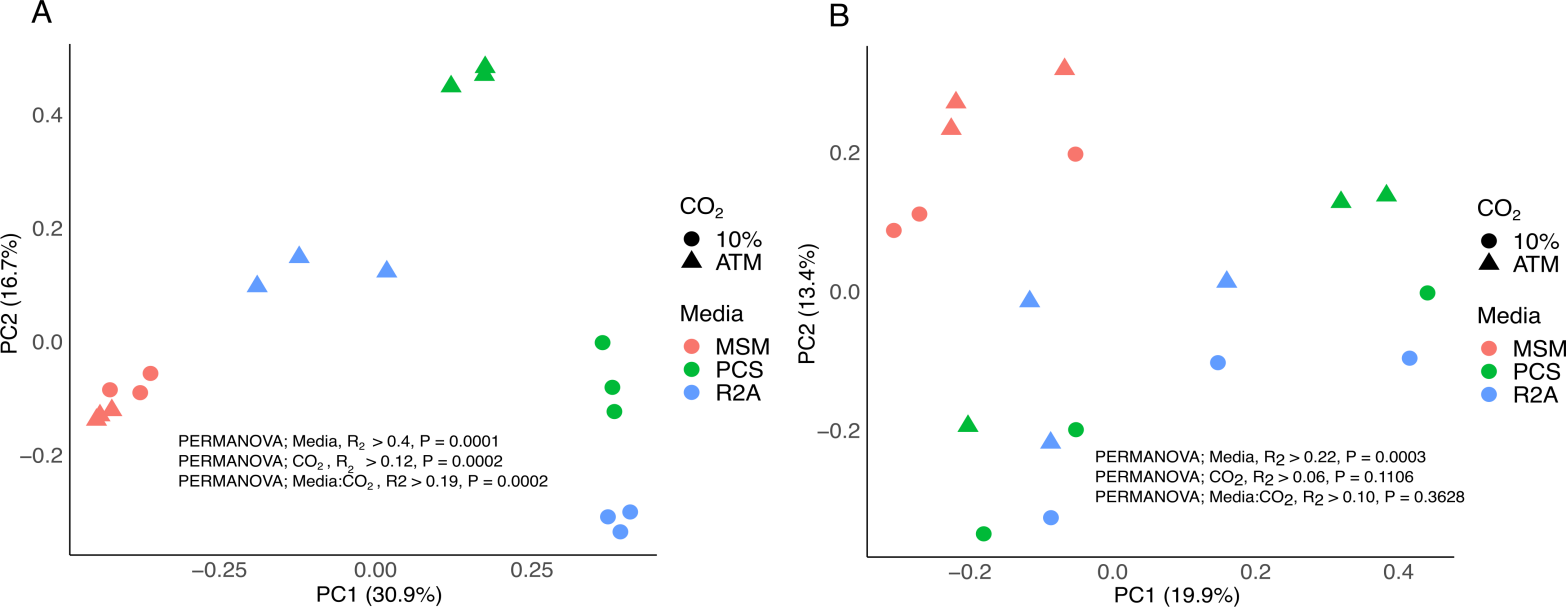
PCoA of the Bray-Curtis bacterial (**A**) and fungi (**B**) community distances between enrichment treatments. Colors indicate culture media, whereas circles represent enrichment cultures with 10% CO_2_ and triangles represent enrichment cultures with atmospheric CO_2_.

The composition of bacterial communities was significantly influenced by the cultivation media (Fig. 2A), with community compositions on MSM, PCS, and R2A media being significantly different from one another (pairwise.adonis; Bray_Curtis, R^2^ = 0.20, *P* < 0.05). More specifically, the ASV clustered at the Phylum level, contributing to discriminate MSM samples from both PCS and R2A samples, comprised 5 phyla, including potential oligotrophic bacteria affiliated to Acidobacteriota and Chloroflexi (Table S8) (54, 55). ASV aggregated at the genus level encompassing *Clostridium* was significantly higher in PCS compared to MSM enrichment cultures (ANCOM-BC, log-fold change > 1.49, *P* < 0.05). The enrichment of cellulolytic *Clostridium* bacteria and the degraded cellulose filter paper strips in PCS cultures suggested that these cultures were efficient for the enrichment of cellulolytic bacteria (56).

The impact of CO_2_ on bacterial community composition was uneven among the three cultivation media. Bacterial communities in R2A and PCS were more responsive to CO_2_ than MSM enrichment cultures (Fig. 2A). The ASV clustered at the genus level, contributing to discriminate samples R2A_CO_2_ and PCS_CO_2_ from all the other samples comprised 17 genera favored by CO_2_ (Table S9). The most responsive taxa include potential anaerobic bacteria affiliated with *Clostridium* sp. and *Macellibacteroides* sp. (ANCOM-BC, log-fold change > 5, *P* < 0.05) (57, 58). Elevated CO_2_ level combined with biomass aggregates in the enrichment cultures was likely favorable to the formation of anaerobic microenvironments in R2A and PCS, while biomass aggregates displayed a lower density in MSM enrichments.

Fungal communities in the enrichment cultures were dominated by Ascomycota, represented by genera encompassing *Ovatospora* and *Chloridium*, followed by *Penicillium*, *Trichoderma,* and *Humicola* (Fig. S3A and B). The composition of the fungal community in MSM enrichments was distinct from R2A and PCS enrichment cultures (Fig. 2B). ASV aggregated at the genus level encompassing *Thermomyces, Tausonia, Mrakia,* and *Metarhizium* was significantly lower in MSM compared to the other enrichment cultures, whereas 12 genera were more abundant in MSM, including *Trichoderma* (Table S10). CO_2_ exposure led to no coherent response at the community level (Fig. 2B), and no significant response was observed at the ASV level (ANCOM-BC, *P* > 0.05).

### Microbial diversity in lignocellulosic substrate

The microbial diversity of the lignocellulosic substrate was analyzed after *P. ostreatus* growth, inoculated with the different microbial enrichment cultures. The species richness decreased significantly between enrichment culture and lignocellulosic substrate treatment for both bacterial and fungal diversity (Table S7).

**Fig 3:**
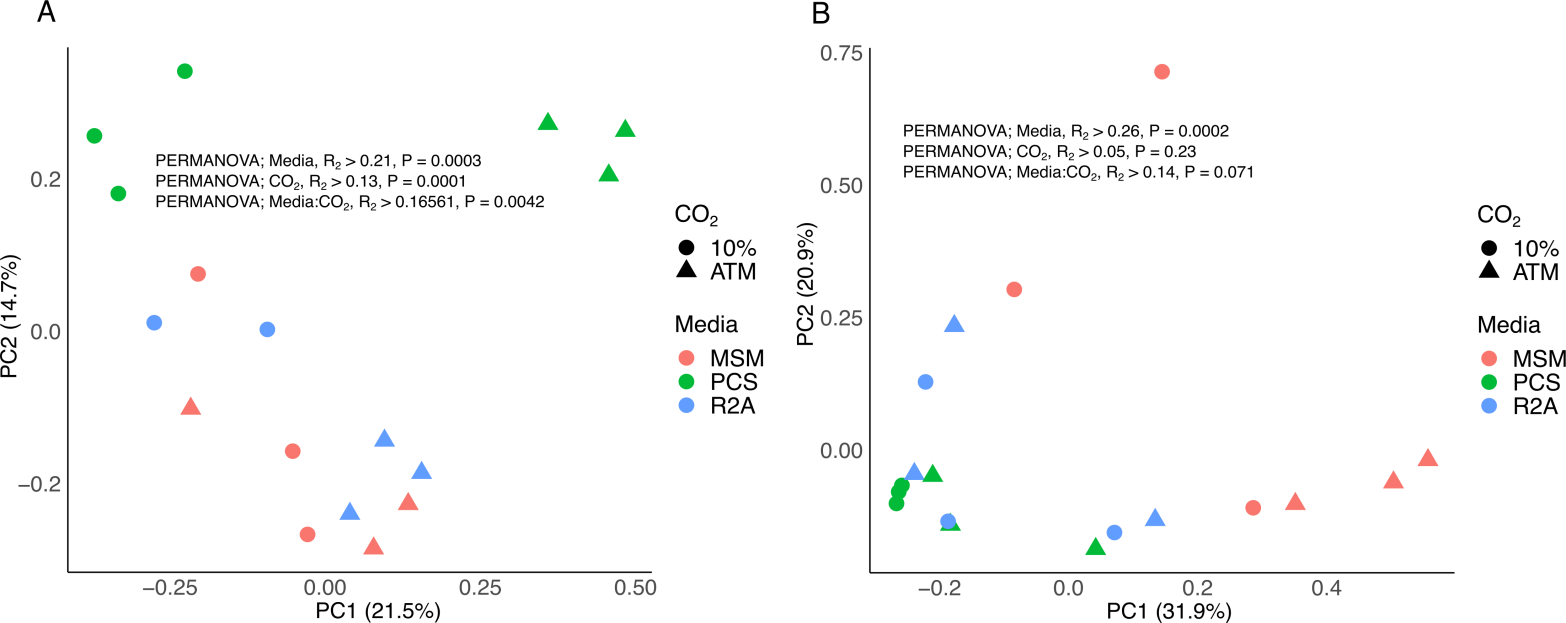
PCoA of the Bray-Curtis bacterial (**A**) and fungal (**B**) communities distances between lignocellulosic substrate treatments. Colors indicate culture media, whereas circles represent enrichment cultures with 10% CO_2_ and triangles represent enrichment cultures with atmospheric CO_2_. The third R2A replicate is missing due to an unsuccessful sequencing reaction.

The CO_2_ treatments, the cultivation media, and the interaction among both factors were significant drivers of bacterial community composition (Fig. 3A). The composition of bacterial communities in lignocellulosic substrate inoculated with MSM enrichment cultures contrasted with those from PCS treatment (pairwise.adonis; Bray_Curtis, R^2^ = 0.17, *P* < 0.05). The relative abundance of ASV affiliated to the genera *Mycobacterium, Flavobacterium, Chryseobacterium, Chitinophaga,* and *Asticcacaulis* was higher in MSM treatment compared to PCS (Table S11). Although the composition of the bacterial communities of R2A was initially different from PCS, they converged toward an indistinguishable community in the lignocellulosic substrate. A legacy effect of CO_2_ was also noticed, in substrates inoculated with PCS_atm and PCS_CO_2_ enrichment cultures, with the relative abundance of aggregated ASV at the genus level encompassing *Youhaiella, Pseudomonas, Pluralibacter,* and *Asticcacaulis* was favored in PCS_CO_2_ (ANCOM-BC; *P* < 0.05).

Fungi were dominated by ASV encompassing the genus *Trichoderma* (Ascomycota) and *Pleurotus* (Basidiomycota) (Fig. S3B). The exclusion of most of the fungal diversity suggested a strong competition between *P. ostreatus* and the other fungi inoculated in the substrate. The cultivation media used during the enrichment stage were a significant driver of fungal community composition (Fig. 3B). ASV affiliated with *Trichoderma* sp. was the most abundant in MSM_atm, whereas *Pleurotus* sp. dominated in substrates inoculated with R2A and PCS enrichment cultures (ANCOM-BC; *P* < 0.05).

### *P. ostreatus* mycelium growth in the lignocellulosic substrate

**Fig 4:**
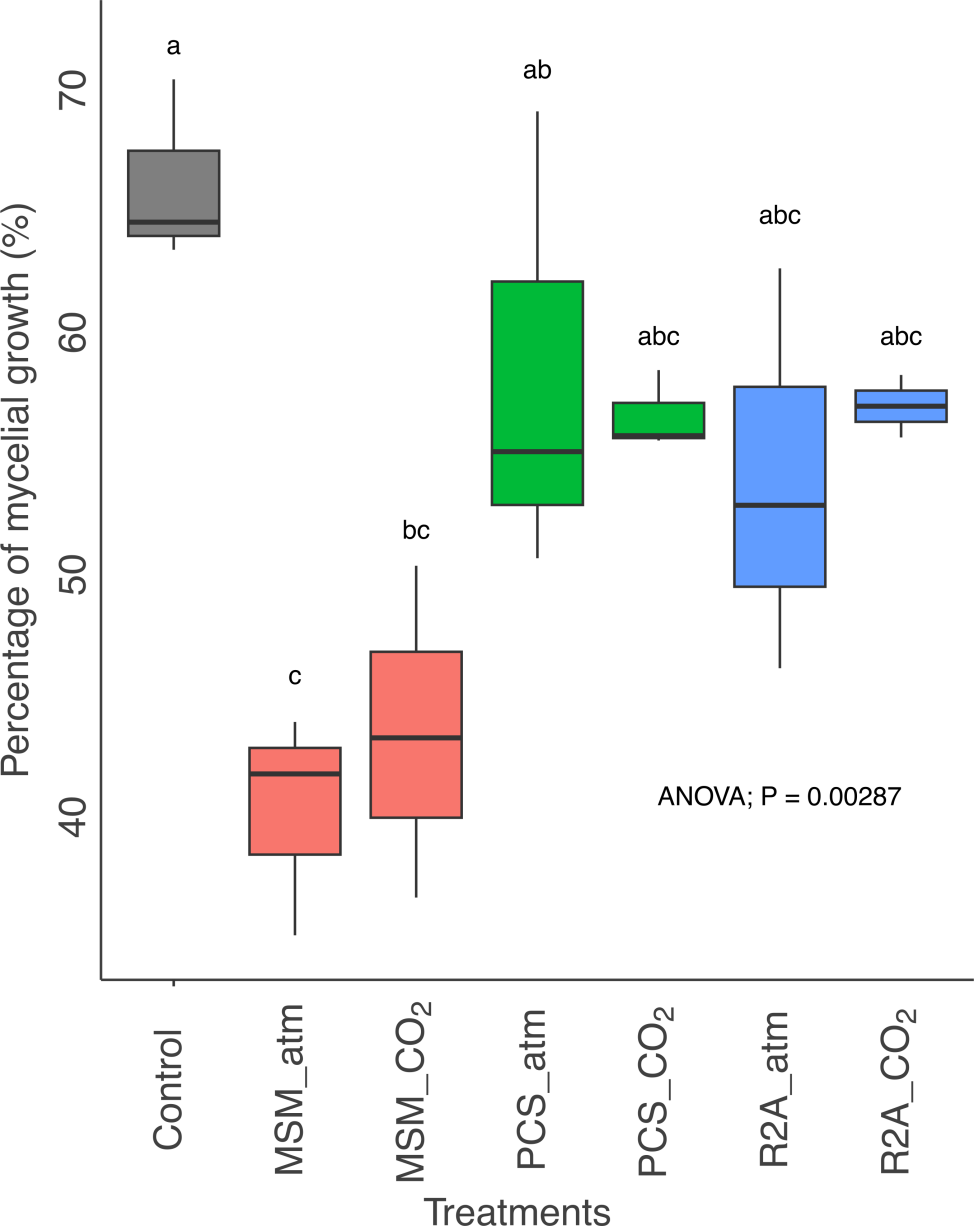
Percentage of *P. ostreatus* mycelial growth in the lignocellulosic substrate according to the different treatments. Control represents substrates inoculated with *P. ostreatus* without microbial inoculum. MSM_atm and MSM_CO_2_ treatment presented a mycelial growth significantly lower compared to the control. Letters differ by Tukey’s test (*P* < 0.05).

The relative abundance of ASV affiliated to *Pleurotus* sp. was proportional to the mycelium growth of *P. ostreatus* assessed by image integration (Pearson correlation, ρ = 0.82, *P* = 5.581e-05) (Table S12). Control substrates without microbial inoculum were visually completely covered with white mycelium after 14 days of incubation, while substrates comprising microbial inoculum presented lower mycelium growth restricted to the upper part of the substrate (Fig. S4). *P. ostreatus* growth was reduced with microbial inoculum, but after three additional weeks of incubation, the fungus achieved the same growth in substrates inoculated with the R2A and PCS enrichment compared to the control. MSM_atm and MSM_CO_2_ treatments presented a lower mycelial growth compared to the control substrates (Fig. 4). Mycelial growth was significantly lower in MSM treatment compared to PCS treatment, but the difference was lost in enrichment culture from the same media exposed to elevated CO_2_ (MSM_CO_2_).

### Covariation of microbial communities and *P. ostreatus* growth performance

**Fig 5:**
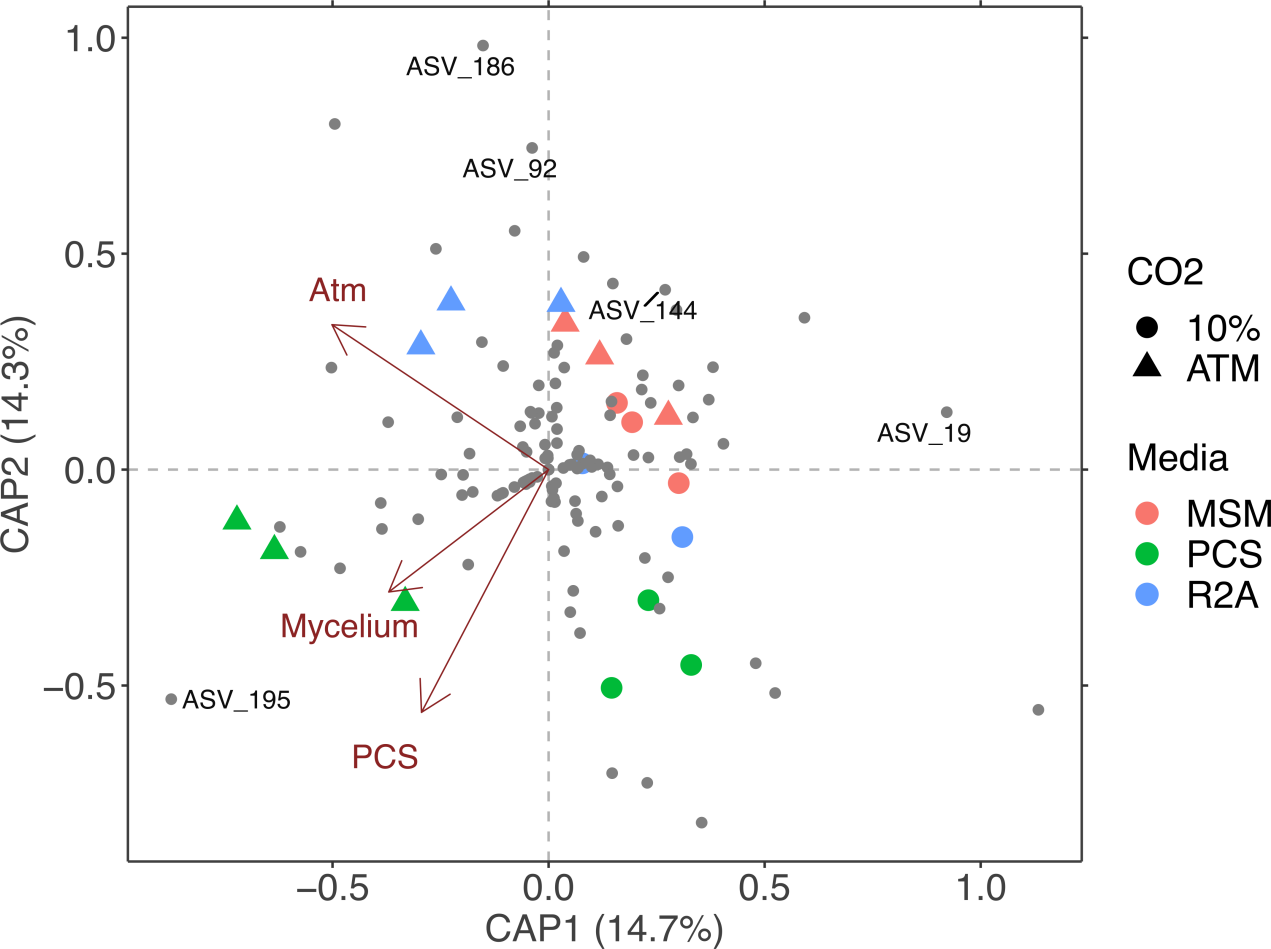
db-RDA based on Bray-Curtis distances showing the response of bacterial community composition, in lignocellulosic substrate treatments, to several parameters (the cultivation media, the headspace, the growth performance of *P. ostreatus,* and the relative proportion of ASVs *Pleurotus* spp. in the different treatments). The mycelium growth (*P* = 0.05), the PCS condition (*P* = 0.01), and the headspace (*P* = 0.05) contributed significantly (38%) to this variation (Bray-Curtis db-RDA, R_2_ adj = 0.24, *P* = 0.001). Four ASVs (numbered 19, 92, 186, 144) are in the opposite direction of the “Mycelium” variable and represent the phylotypes *Pseudomonas* sp., *Shinella* sp., *Rhizobium* sp., *Asticcacaulis* sp., respectively, while ASV numbered 195 is in the same direction as the “Mycelium” variable and represents the phylotype *Brevundimonas* sp. The different ASVs are represented with small gray dots. Colors indicate culture media, whereas circles represent enrichment cultures with 10% CO_2_. Arrows indicate significant variables (Mycelium; the growth performance of *P. ostreatus*, Atm; the headspace, and PCS; the PCS medium).

**Fig 6:**
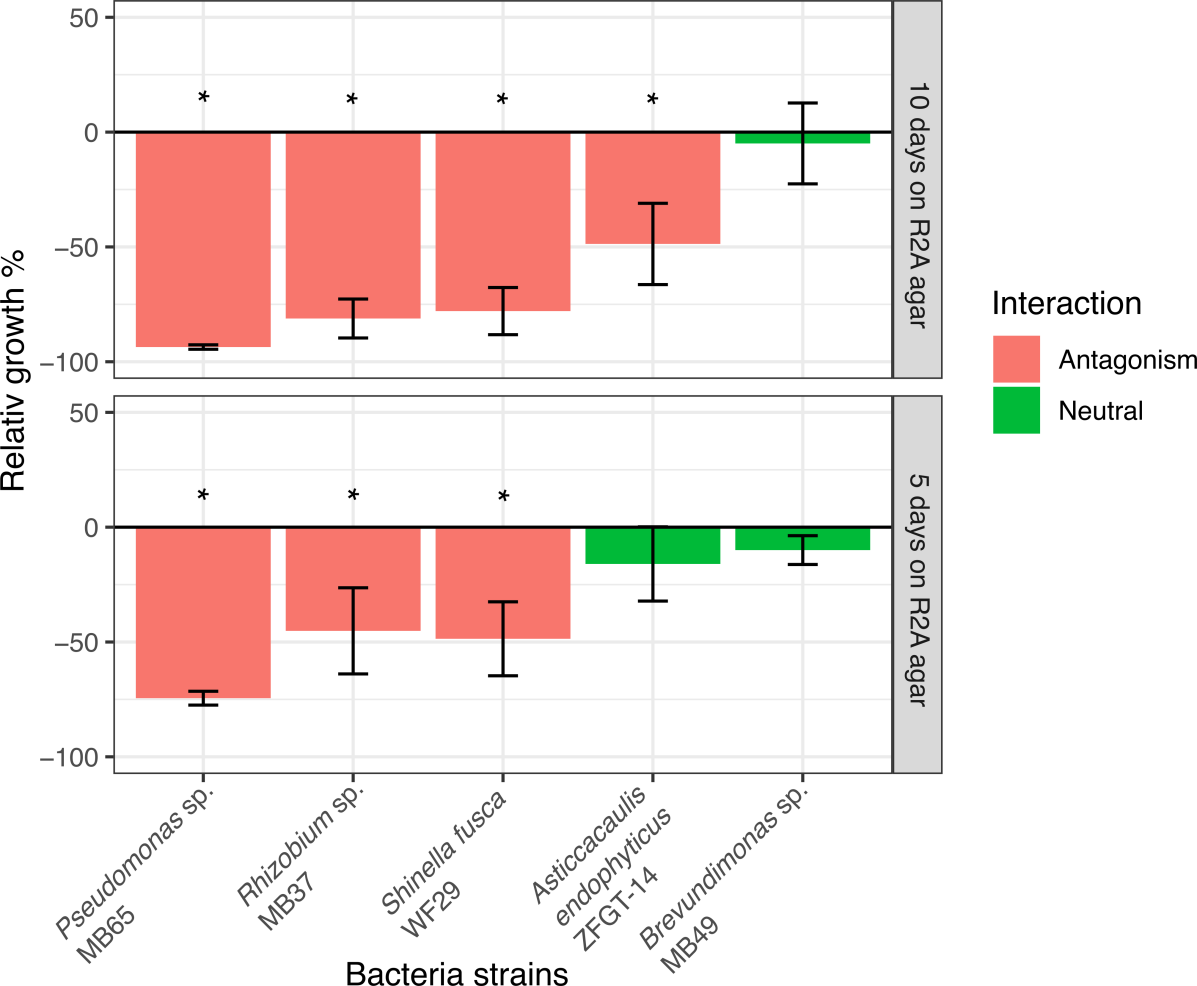
Growth effects of bacteria strains on mycelial growth of *P. ostreatus*. The relative mycelium growth of *P. ostreatus* co-cultured with bacterial isolates (retrieved from a culture collection) on Petri dishes containing R2A agar relative to *P. ostreatus* mycelium alone without bacteria (control) was measured after 5 and 10 days of incubation. Mycelium growth areas were measured after 4 and 8 days for the *in vitro* confrontation with *Brevundimonas* sp. * Significantly different compared to control, ANOVA, *P* < 0.05. Interactions were considered as antagonists if *P* < 0.05 and relative growth < 0, and considered as neutral if *P* > 0.05.

The observed variance in growth rates of *P. ostreatus* across the different treatments (Fig. 4) could be ascribed to dissimilarities among bacterial community composition previously described. The CO_2_ concentration in the headspace of enrichment cultures, the PCS medium, and the growth performance of *P. ostreatus* were the most significant features explaining the variation of the bacterial community structure in lignocellulosic substrate, with the PCS medium showing a positive correlation with the growth performance of *P. ostreatus* (Fig. 5). These results suggest that the composition of bacterial community in PCS enrichment culture was the most compatible with the growth performance of *P. ostreatus*. More specifically, the relative abundance of *Brevundimonas* sp. was significantly higher in PCS treatment compared to the other treatments (ANCOM-BC, log-fold change > 3, *P* < 0.05). Covariation of mycelium growth with the distribution of ASV 195 (*Brevundimonas* sp.) suggests a neutral or auxiliary effect of the bacterium (Fig. 5). The distribution of potential antagonistic phylotypes affiliated with *Pseudomonas* sp. (ASV 19), *Asticcacaulis* sp. (ASV 144), *Shinella* sp. (ASV 92), and *Rhizobium* sp. (ASV 186) displayed the opposite pattern (Fig. 5). Conservation of the potential antagonistic, neutral, or auxiliary effect of phylotypes was further tested with bacterial isolates affiliated to the same genera of available in public and in-house collections. The mycelial growth surface of *P. ostreatus* was significantly reduced in the presence of isolates affiliated to *Pseudomonas* sp. MB65*, Rhizobium* sp. MB37, *Asticcacaulis* sp. ZFGT-14, and *Shinella* sp. WF29 after 10 days on R2A agar (Fig. 6).

In contrast, the growth of *P. ostreatus* was neither reduced nor favored in the presence of the isolate *Brevundimonas* sp. MB49 for both *in vitro* (Fig. 6) and in lignocellulosic substrate-based bioassays (ANOVA, *P* > 0.05) (Tables S13 and S14). For the latter, bacterial abundance was significantly higher within the substrate area without mycelium or within the growing mycelium compared to the bacterial abundance assessed in the more senescent mycelial region (Fig. S5; Table S15). Together, these observations support conservation of neutral and antagonist bacteria-fungi interactions at the genus and species levels.

## DISCUSSION

Although the diversity of microbial communities in the mycelium and in the growing environment of *P. ostreatus* has been studied (35, 59), the establishment of a compatible community beneficial to the growth of the fungus in lignocellulosic biomass appeared complex. Microbial communities were manipulated through enrichment by using different culture media and by modifying the CO_2_ atmosphere. In the latter, headspace was modified to promote complementary functions related to carbon fixation metabolism absent in mushrooms. Formulation of cultivation media, comprising various carbon amendments, ranging from recalcitrant (MSM) to labile carbon sources (PCS and R2A), exerted a greater effect on microbial diversity when compared to CO_2_ favorable for chemolithoautotrophic growth of a few members of the microbial communities.

*P. ostreatus* growth was less efficient in lignocellulosic substrate co-inoculated with MSM enrichment cultures targeting saprotrophic communities than inoculated alone. Lignocellulosic biomass, as the sole energy and carbon source in MSM enrichment, likely accounted for the higher species richness compared to PCS and R2A media, comprising more labile nutrients, leading to the enrichment of dominant fast-growing micro-organisms (60). The richness of microbial enrichment in MSM likely induced a strong competition due to resource partitioning involving cheater species competing for the labile carbon released by the fungus (19). MSM enrichment conditions, close to those of the lignocellulosic environment, also enabled the MSM community to be stable and thus to survive and grow once in the substrate, key for niche occupancy success (61). Finally, oligotrophic phyla in the MSM enriched community, affiliated to Acidobacteriota and Chloroflexi, likely increased competition with the fungus due to their high substrate affinity (62, 63). Together, the functional redundancy related to substrate degradation and the efficient niche occupancy of MSM enrichments promoted competition between the inoculum and *P. ostreatus*, leading to a reduced growth of the fungus.

The recalcitrant nature of lignocellulosic substrates is conducive to the division of metabolic labor and niche partitioning (61). Carbon labile sources integrated into PCS and R2A likely enriched carbon-degrading functions beyond saprotroph metabolism and could have improved consortia-*P. ostreatus* compatibility compared to MSM enrichments. However, substrate inoculated with R2A and PCS communities, with functions including cellulose breakdown for the latter, did not enhance fungal growth when compared to the control, suggesting that enzymatic synergy is not sufficient to enhance fungal biomass (27, 64). R2A and PCS communities also presented a lower microbial diversity compared to MSM’s communities; this trade-off in diversity could have lowered the likelihood of auxiliary functions. Thus, nutrient and CO_2_ manipulation of the enrichment media was not an effective strategy to drive a consortium beneficial to *P. ostreatus* growth. Taken together, these results suggested that functions linked to primary carbon metabolism are not those primarily involved in *P. ostreatus* promotion.

Analysis of microbial communities led to the identification of potential antagonistic and compatible bacteria and fungi for *P. ostreatus*. The reduced growth of the fungus in substrates inoculated with MSM enrichment could be a consequence of a strong competition with the dominant fungi *Trichoderma* spp., initially more abundant in the MSM enrichment compared to other enrichments. These fungi are efficient competitors, capable of rapidly colonizing lignocellulosic substrates. Known as green-mold disease, *Trichoderma* sp. is an issue in mushroom farms, causing significant loss in edible fungi crops (65). This taxon was also detected in R2A and PCS enrichments, ranking within the five most dominant fungal taxa in the enrichment cultures. Abundance of *Trichoderma* spp. alone was thus not sufficient to explain the variation of *P. ostreatus* growth performances. Specific bacteria likely contributed to *P. ostreatus* growth inhibition, as supported by correlations between *P. ostreatus* growth and the distribution of certain bacterial taxa in multivariate analyses. The causality of these correlations was supported by an isolate affiliated with *Asticcacaulis* sp.. The bacterial abundance of this genus was significantly higher in the MSM treatments, both in enrichment cultures and in the substrate, compared to other treatments. The potential antagonistic activity of *Asticcacaulis* spp. against *P. ostreatus* was validated *in vitro* along with isolates affiliated with *Pseudomonas* sp. MB65*, Rhizobium* sp. MB37, and *Shinella* sp. WF29. Conversely, indirect benefits for *P. ostreatus* may have contributed to higher performance in PCS and R2A through the deleterious effects of bacteria exerting antagonistic interaction with *Trichoderma* sp. In a previous study, bacteria of the *Bacillus* genus have demonstrated their ability to selectively inhibit the growth of *Trichoderma* sp. without affecting that of *P. ostreatus*, through the production of fengycin, an antifungal peptide (66). Finally, only one taxon, *Brevundimonas* sp., was compatible with *P. ostreatus*. ASV assigned in the genus *Brevundimonas* was more abundant in substrate inoculated with PCS compared to other treatments and *Brevundimonas* sp. MB49 was the sole isolate displaying no antagonistic effect in the *in vitro* confrontation test. *Brevundimonas* sp. strain was identified as a *P. ostreatus* growth-promoting bacterium in a previous report (67), but that trait is not conserved at the genus level, according to the *in vitro* and lignocellulosic-based assays conducted with *Brevundimonas* sp. MB49. Finally, interactions appeared to be driven by a trade-off between compatibility and colonization constraints imposed by unbalanced bacterial and fungal inocula. This is supported by neutral or negative impacts of *P. ostreatus* on the growth of *Brevundimonas* sp. MB49 in lignocellulosic-substrate-based assays. The difference in bacterial abundance between growing and senescent mycelium suggested that compatible inter-kingdom interactions could be constrained by the growth stage of the mycelium (68).

Microbial communities became more homogeneous in the lignocellulosic substrate across the different trials due to a filtering effect induced by environmental conditions and *P. ostreatus* itself. Indeed, bacteria compatible with fungal growth, such as *Brevundimonas* sp., were more abundant in trials with robust fungal growth, suggesting a selection by the fungus for less inhibitory bacterial communities (69). This is in line with previous findings that *P. ostreatus* can modify and select its surrounding microbial communities during growth (70), leading to a microbial succession (15). Due to its ability to influence microbial diversity, the fungus may be considered less as an individual within a community—where interactions are examined on a pairwise basis—but rather as a host, with the hyphosphere environment functioning similarly to the plant’s rhizosphere (71). According to this concept, the establishment of a self-assembled consortium could be achieved through directed evolution. Co-culture of *P. ostreatus* with bacteria would be more conducive to auxiliary enrichments (72), whose beneficial functions are difficult to select using selective media. Key organisms that contribute to fungal growth promotion, along with the characterization of their auxiliary traits and their interplay with other species, could be further identified through isolation and assembly into synthetic communities (73).

### Conclusion

Our results indicate that enrichment cultures only containing recalcitrant carbon sources are less compatible with *P. ostreatus* than enrichment cultures containing a combination of recalcitrant and labile carbon sources. The growth of *P. ostreatus* was nevertheless most efficient in the absence of microbial inoculum. Carbon degradation functions are not the main drivers of microbial communities beneficial to the growth of *P. ostreatus*. Other functions beyond the primary metabolism of carbon could be involved in its promotion. This knowledge will drive further studies on the assembly of compatible bacterial and fungal communities, as well as identifying ecological traits involved in fungal growth promotion. Ultimately, these future directions are expected to stabilize and optimize fungal biomass production and lignocellulosic feedstock pretreatments for bioproducts and bioenergy production.

## Data Availability

All raw data generated by this study have been deposited on NCBI under BioProject PRJNA1188180. This BioProject includes amplicon sequencing data of enrichment cultures and lignocellulosic substrates. The ASV table is available in the supplemental material. All code used for data processing and analyses is available in GitHub: https://github.com/LaboPC/Pleurotus-bacteria-enrichment_MarieBonduelle_2024

## SUPPLEMENTAL MATERIAL

Supplemental figures aem.00898-25-s0001.pdf Figures S1 to S5.

Supplemental tables aem.00898-25-s0002.xlsx Tables S2 to S15.

ASM does not own the copyrights to Supplemental Material that may be linked to, or accessed through, an article. The authors have granted ASM a non-exclusive, world-wide license to publish the Supplemental Material files. Please contact the corresponding author directly for reuse.

## References

[1] Frey-Klett P, Burlinson P, Deveau A, Barret M, Tarkka M, Sarniguet A. 2011. Bacterial-fungal interactions: hyphens between agricultural, clinical, environmental, and food microbiologists. Microbiol Mol Biol Rev 75:583–609. doi:10.1128/MMBR.00020-11 22126995 PMC3232736

[2] Deveau A, Brulé C, Palin B, Champmartin D, Rubini P, Garbaye J, Sarniguet A, Frey‐Klett P. 2010. Role of fungal trehalose and bacterial thiamine in the improved survival and growth of the ectomycorrhizal fungus Laccaria bicolor S238N and the helper bacterium Pseudomonas fluorescens BBc6R8. Environ Microbiol Rep 2:560–568. doi:10.1111/j.1758-2229.2010.00145.x 23766226

[3] Mela F, Fritsche K, de Boer W, van Veen JA, de Graaff LH, van den Berg M, Leveau JHJ. 2011. Dual transcriptional profiling of a bacterial/fungal confrontation: Collimonas fungivorans versus Aspergillus niger. ISME J 5:1494–1504. doi:10.1038/ismej.2011.29 21614084 PMC3160687

[4] Hildebrandt U, Ouziad F, Marner FJ, Bothe H. 2006. The bacterium Paenibacillus validus stimulates growth of the arbuscular mycorrhizal fungus Glomus intraradices up to the formation of fertile spores. FEMS Microbiol Lett 254:258–267. doi:10.1111/j.1574-6968.2005.00027.x 16445754

[5] Riedlinger J, Schrey SD, Tarkka MT, Hampp R, Kapur M, Fiedler H-P. 2006. Auxofuran, a novel metabolite that stimulates the growth of fly agaric, is produced by the mycorrhiza helper bacterium Streptomyces Strain AcH 505. Appl Environ Microbiol 72:3550–3557. doi:10.1128/AEM.72.5.3550-3557.2006 16672502 PMC1472321

[6] Zhou J, Wang J, Zhou Y, Liu K, Lu Y, Zhu L, Chen X. 2023. Microbial community structure and interactions between Aspergillus oryzae and bacteria in traditional solid-state fermentation of Jiangqu. Food Microbiol 116:104346. doi:10.1016/j.fm.2023.104346 37689429

[7] Sánchez C. 2009. Lignocellulosic residues: biodegradation and bioconversion by fungi. Biotechnol Adv 27:185–194. doi:10.1016/j.biotechadv.2008.11.001 19100826

[8] Jönsson L, Palmqvist E, Nilvebrant NO, Hahn-Hägerdal B. 1998. Detoxification of wood hydrolysates with laccase and peroxidase from the white-rot fungus Trametes versicolor. Appl Microbiol Biotechnol 49:691–697. doi:10.1007/s002530051233

[9] Grimm D, Sonntag E, Rahmann G. 2024. Evaluation of different pasteurization and sterilization methods for oyster mushroom substrates. J Microb Biotech Food Sci 13:e10428. doi:10.55251/jmbfs.10428

[10] Zhao X, Zhang L, Liu D. 2012. Biomass recalcitrance. Part I: the chemical compositions and physical structures affecting the enzymatic hydrolysis of lignocellulose. Biofuels Bioprod Bioref 6:465–482. doi:10.1002/bbb.1331

[11] Cragg SM, Beckham GT, Bruce NC, Bugg TDH, Distel DL, Dupree P, Etxabe AG, Goodell BS, Jellison J, McGeehan JE, McQueen-Mason SJ, Schnorr K, Walton PH, Watts JEM, Zimmer M. 2015. Lignocellulose degradation mechanisms across the tree of life. Curr Opin Chem Biol 29:108–119. doi:10.1016/j.cbpa.2015.10.018 26583519 PMC7571853

[12] Zervakis GI, Ntougias S, Gargano ML, Besi MI, Polemis E, Typas MA, Venturella G. 2014. A reappraisal of the Pleurotus eryngii complex - new species and taxonomic combinations based on the application of a polyphasic approach, and an identification key to Pleurotus taxa associated with Apiaceae plants. Fungal Biol 118:814–834. doi:10.1016/j.funbio.2014.07.001 25209640

[13] Febnteh E, Anjah G, Kinge T, Ambebe T. 2023. Abundance and diversity of pleurotus species and host trees for sustainable management in ngel-nyaki montane forest ecosystem. East African Scholars Multidisciplinary Bulletin 6. doi:10.36349/easjmb.2023.v06i05.001

[14] Del Cerro C, Erickson E, Dong T, Wong AR, Eder EK, Purvine SO, Mitchell HD, Weitz KK, Markillie LM, Burnet MC, Hoyt DW, Chu RK, Cheng J-F, Ramirez KJ, Katahira R, Xiong W, Himmel ME, Subramanian V, Linger JG, Salvachúa D. 2021. Intracellular pathways for lignin catabolism in white-rot fungi. Proc Natl Acad Sci USA 118:e2017381118. doi:10.1073/pnas.2017381118 33622792 PMC7936344

[15] Chen L, Yan M, Qian X, Yang Z, Xu Y, Wang T, Cao J, Sun S. 2022. Bacterial community composition in the growth process of Pleurotus eryngii and growth-promoting abilities of isolated bacteria. Front Microbiol 13:787628. doi:10.3389/fmicb.2022.787628 35173699 PMC8842659

[16] Carrasco J, Preston GM. 2020. Growing edible mushrooms: a conversation between bacteria and fungi. Environ Microbiol 22:858–872. doi:10.1111/1462-2920.14765 31361932

[17] Adamski M, Pietr SJ. 2019. Biodiversity of bacteria associated with eight Pleurotus ostreatus (Fr.) P. Kumm. strains from Poland, Japan and the USA. Pol J Microbiol 68:71–81. doi:10.21307/pjm-2019-009 31050255 PMC7256699

[18] Møller J, Miller M, Kjøller A. 1999. Fungal–bacterial interaction on beech leaves: influence on decomposition and dissolved organic carbon quality. Soil Biol Biochem 31:367–374. doi:10.1016/S0038-0717(98)00138-2

[19] Wang C, Kuzyakov Y. 2024. Mechanisms and implications of bacterial-fungal competition for soil resources. ISME J 18:wrae073. doi:10.1093/ismejo/wrae073 38691428 PMC11104273

[20] Ritz K. 1995. Growth responses of some soil fungi to spatially heterogeneous nutrients. FEMS Microbiol Ecol 16:269–280. doi:10.1111/j.1574-6941.1995.tb00291.x

[21] Schneider T, Gerrits B, Gassmann R, Schmid E, Gessner MO, Richter A, Battin T, Eberl L, Riedel K. 2010. Proteome analysis of fungal and bacterial involvement in leaf litter decomposition. Proteomics 10:1819–1830. doi:10.1002/pmic.200900691 20198641

[22] Mille‐Lindblom C, Fischer H, J. Tranvik L. 2006. Antagonism between bacteria and fungi: substrate competition and a possible tradeoff between fungal growth and tolerance towards bacteria. Oikos 113:233–242. doi:10.1111/j.2006.0030-1299.14337.x

[23] Stopnisek N, Zühlke D, Carlier A, Barberán A, Fierer N, Becher D, Riedel K, Eberl L, Weisskopf L. 2016. Molecular mechanisms underlying the close association between soil Burkholderia and fungi. ISME J 10:253–264. doi:10.1038/ismej.2015.73 25989372 PMC4681855

[24] Cortes-Tolalpa L, Salles JF, van Elsas JD. 2017. Bacterial synergism in lignocellulose biomass degradation - complementary roles of degraders as influenced by complexity of the carbon source. Front Microbiol 8:1628. doi:10.3389/fmicb.2017.01628 29067002 PMC5641323

[25] Lee JA, Baugh AC, Shevalier NJ, Strand B, Stolyar S, Marx CJ. 2021. Cross-feeding of a toxic metabolite in a synthetic lignocellulose-degrading microbial community. Microorganisms 9:321. doi:10.3390/microorganisms9020321 33557371 PMC7914493

[26] Kumari S, Naraian R. 2021. Enhanced growth and yield of oyster mushroom by growth-promoting bacteria Glutamicibacter arilaitensis MRC119. J Basic Microbiol 61:45–54. doi:10.1002/jobm.202000379 33347662

[27] Torres-Ruiz E, Sanchez JE, Guillen-Navarro GK, Ramos-Perez DG, Royse DJ. 2016. Microbial promoters of mycelial growth, fruiting and production of Pleurotus ostreatus. Sydowia. doi:10.12905/0380.sydowia68-2016-0151:151-161

[28] Kim MK, Math RK, Cho KM, Shin KJ, Kim JO, Ryu JS, Lee YH, Yun HD. 2008. Effect of Pseudomonas sp. P7014 on the growth of edible mushroom Pleurotus eryngii in bottle culture for commercial production. Bioresour Technol 99:3306–3308. doi:10.1016/j.biortech.2007.06.039 17698350

[29] Suarez C, Ratering S, Weigel V, Sacharow J, Bienhaus J, Ebert J, Hirz A, Rühl M, Schnell S. 2020. Isolation of bacteria at different points of Pleurotus ostreatus cultivation and their influence in mycelial growth. Microbiol Res 234:126393. doi:10.1016/j.micres.2019.126393 32036274

[30] Cho YS, Kim JS, Crowley DE, Cho BG. 2003. Growth promotion of the edible fungus Pleurotus ostreatus by fluorescent pseudomonads. FEMS Microbiol Lett 218:271–276. doi:10.1016/S0378-1097(02)01144-8 12586403

[31] Hoppe B, Kahl T, Karasch P, Wubet T, Bauhus J, Buscot F, Krüger D. 2014. Network analysis reveals ecological links between N-fixing bacteria and wood-decaying fungi. PLoS One 9:e88141. doi:10.1371/journal.pone.0088141 24505405 PMC3914916

[32] Chen S, Qiu C, Huang T, Zhou W, Qi Y, Gao Y, Shen J, Qiu L. 2013. Effect of 1-aminocyclopropane-1-carboxylic acid deaminase producing bacteria on the hyphal growth and primordium initiation of Agaricus bisporus. Fungal Ecol 6:110–118. doi:10.1016/j.funeco.2012.08.003

[33] Kang YM, Cho KM. 2014. Identification of auxin from Pseudomonas sp. P7014 for the rapid growth of Pleurotus eryngii mycelium. Korean J Microbiol 50:15–21. doi:10.7845/kjm.2014.3076

[34] Orban A, Jerschow JJ, Birk F, Suarez C, Schnell S, Rühl M. 2023. Effect of bacterial volatiles on the mycelial growth of mushrooms. Microbiol Res 266:127250. doi:10.1016/j.micres.2022.127250 36343596

[35] Paul C, Roy T, Singh K, Maitra M, Das N. 2023. Study of growth-improving and sporophore-inducing endobacteria isolated from Pleurotus pulmonarius. World J Microbiol Biotechnol 39:349. doi:10.1007/s11274-023-03776-0 37857876

[36] Li R, Zhang Q, Chen Y, Gao Y, Yang Y, Liu Q, Kong W, Chai H, Sun B, Li Y, Qiu L. 2025. The mechanism of ammonia-assimilating bacteria promoting the growth of oyster mushrooms (Pleurotus ostreatus). J Fungi (Basel) 11:130. doi:10.3390/jof11020130 39997424 PMC11856247

[37] Folman LB, Klein Gunnewiek PJA, Boddy L, de Boer W. 2008. Impact of white-rot fungi on numbers and community composition of bacteria colonizing beech wood from forest soil. FEMS Microbiol Ecol 63:181–191. doi:10.1111/j.1574-6941.2007.00425.x 18199083

[38] Wongwilaiwalin S, Rattanachomsri U, Laothanachareon T, Eurwilaichitr L, Igarashi Y, Champreda V. 2010. Analysis of a thermophilic lignocellulose degrading microbial consortium and multi-species lignocellulolytic enzyme system. Enzyme Microb Technol 47:283–290. doi:10.1016/j.enzmictec.2010.07.013

[39] Baril X, Durand AA, Srei N, Lamothe S, Provost C, Martineau C, Dunfield K, Constant P. 2022. The biological sink of atmospheric H2 is more sensitive to spatial variation of microbial diversity than N2O and CO2 emissions in a winter cover crop field trial. Sci Total Environ 821:153420. doi:10.1016/j.scitotenv.2022.153420 35092770

[40] Schneider CA, Rasband WS, Eliceiri KW. 2012. NIH Image to ImageJ: 25 years of image analysis. Nat Methods 9:671–675. doi:10.1038/nmeth.2089 22930834 PMC5554542

[41] Edwards JE, Huws SA, Kim EJ, Kingston-Smith AH. 2007. Characterization of the dynamics of initial bacterial colonization of nonconserved forage in the bovine rumen. FEMS Microbiol Ecol 62:323–335. doi:10.1111/j.1574-6941.2007.00392.x 17941835

[42] Martin KJ, Rygiewicz PT. 2005. Fungal-specific PCR primers developed for analysis of the ITS region of environmental DNA extracts. BMC Microbiol 5:28. doi:10.1186/1471-2180-5-28 15904497 PMC1156903

[43] Saavedra-Lavoie J, de la Porte A, Piché-Choquette S, Guertin C, Constant P. 2020. Biological H_2_ and CO oxidation activities are sensitive to compositional change of soil microbial communities. Can J Microbiol 66:263–273. doi:10.1139/cjm-2019-0412 31999470

[44] RStudio. 2022. RStudio: integrated development for R. RStudio. Available from: http://www.rstudio.com

[45] Marcel M. 2011. Cutadapt removes adapter sequences from high-throughput sequencing reads. EMBnet J 17:10. doi:10.14806/ej.17.1.200

[46] Callahan BJ, McMurdie PJ, Rosen MJ, Han AW, Johnson AJA, Holmes SP. 2016. DADA2: high-resolution sample inference from Illumina amplicon data. Nat Methods 13:581–583. doi:10.1038/nmeth.3869 27214047 PMC4927377

[47] Quast C, Pruesse E, Yilmaz P, Gerken J, Schweer T, Yarza P, Peplies J, Glöckner FO. 2013. The SILVA ribosomal RNA gene database project: improved data processing and web-based tools. Nucleic Acids Res 41:D590–D596. doi:10.1093/nar/gks1219 23193283 PMC3531112

[48] Abarenkov K, Nilsson RH, Larsson KH, Taylor AFS, May TW, Frøslev TG, Pawlowska J, Lindahl B, Põldmaa K, Truong C, et al.. 2024. The UNITE database for molecular identification and taxonomic communication of fungi and other eukaryotes: sequences, taxa and classifications reconsidered. Nucleic Acids Res 52:D791–D797. doi:10.1093/nar/gkad1039 37953409 PMC10767974

[49] Bokulich NA, Subramanian S, Faith JJ, Gevers D, Gordon JI, Knight R, Mills DA, Caporaso JG. 2013. Quality-filtering vastly improves diversity estimates from Illumina amplicon sequencing. Nat Methods 10:57–59. doi:10.1038/nmeth.2276 23202435 PMC3531572

[50] Hsieh TC, Ma KH, Chao A. 2016. iNEXT: an R package for rarefaction and extrapolation of species diversity ( h ill numbers). Methods Ecol Evol 7:1451–1456. doi:10.1111/2041-210X.12613

[51] Oksanen J, Blanchet FG, Kindt R, Legendre P, Minchin PR, O’hara R, Wagner H. 2013. vegan: community ecology package. R package version 2

[52] Lin H, Peddada SD. 2020. Analysis of compositions of microbiomes with bias correction. Nat Commun 11:3514. doi:10.1038/s41467-020-17041-7 32665548 PMC7360769

[53] Blanchet FG, Legendre P, Borcard D. 2008. Forward selection of explanatory variables. Ecology 89:2623–2632. doi:10.1890/07-0986.1 18831183

[54] Fierer N, Bradford MA, Jackson RB. 2007. Toward an ecological classification of soil bacteria. Ecology 88:1354–1364. doi:10.1890/05-1839 17601128

[55] Dragone NB, Hoffert M, Strickland MS, Fierer N. 2024. Taxonomic and genomic attributes of oligotrophic soil bacteria. ISME Commun 4:ycae081. doi:10.1093/ismeco/ycae081 38988701 PMC11234899

[56] Cui J, Mai G, Wang Z, Liu Q, Zhou Y, Ma Y, Liu C. 2019. Metagenomic insights into a cellulose-rich niche reveal microbial cooperation in cellulose degradation. Front Microbiol 10:618. doi:10.3389/fmicb.2019.00618 30984144 PMC6447707

[57] Collins M, Lawson P, Willems A, Cordoba J, Fernandez-Garayzabal J, Garcia P, Farrow J. 1994. The phylogeny of the genus Clostridium: proposal of five new genera and eleven new species combinations. Int J Syst Evol Microbiol 44:812–826. doi:10.1099/00207713-44-4-812 7981107

[58] Jabari L, Gannoun H, Cayol JL, Hedi A, Sakamoto M, Falsen E, Ohkuma M, Hamdi M, Fauque G, Ollivier B, Fardeau ML. 2012. Macellibacteroides fermentans gen. nov., sp. nov., a member of the family Porphyromonadaceae isolated from an upflow anaerobic filter treating abattoir wastewaters. Int J Syst Evol Microbiol 62:2522–2527. doi:10.1099/ijs.0.032508-0 22180609

[59] Vajna B, Nagy A, Sajben E, Manczinger L, Szijártó N, Kádár Z, Bordás D, Márialigeti K. 2010. Microbial community structure changes during oyster mushroom substrate preparation. Appl Microbiol Biotechnol 86:367–375. doi:10.1007/s00253-009-2371-3 19967354

[60] Gladden JM, Eichorst SA, Hazen TC, Simmons BA, Singer SW. 2012. Substrate perturbation alters the glycoside hydrolase activities and community composition of switchgrass-adapted bacterial consortia. Biotechnol Bioeng 109:1140–1145. doi:10.1002/bit.24388 22125273

[61] Jiménez DJ, Dini-Andreote F, DeAngelis KM, Singer SW, Salles JF, van Elsas JD. 2017. Ecological insights into the dynamics of plant biomass-degrading microbial consortia. Trends Microbiol 25:788–796. doi:10.1016/j.tim.2017.05.012 28648267

[62] Lauro FM, McDougald D, Thomas T, Williams TJ, Egan S, Rice S, DeMaere MZ, Ting L, Ertan H, Johnson J, Ferriera S, Lapidus A, Anderson I, Kyrpides N, Munk AC, Detter C, Han CS, Brown MV, Robb FT, Kjelleberg S, Cavicchioli R. 2009. The genomic basis of trophic strategy in marine bacteria. Proc Natl Acad Sci USA 106:15527–15533. doi:10.1073/pnas.0903507106 19805210 PMC2739866

[63] Noell SE, Giovannoni SJ. 2019. SAR11 bacteria have a high affinity and multifunctional glycine betaine transporter. Environ Microbiol 21:2559–2575. doi:10.1111/1462-2920.14649 31090982

[64] Haidar R, Compant S, Robert C, Antonielli L, Yacoub A, Grélard A, Loquet A, Brader G, Guyoneaud R, Attard E, Rey P. 2024. Two Paenibacillus spp. strains promote grapevine wood degradation by the fungus Fomitiporia mediterranea: from degradation experiments to genome analyses. Sci Rep 14:15779. doi:10.1038/s41598-024-66620-x 38982270 PMC11233627

[65] Hatvani L, Kocsubé S, Manczinger L, Antal Z, Szekeres A, Druzhinina IS, Vágvölgyi C. 2008. The green mould disease global threat to the cultivation of oyster mushroom (*Pleurotus ostreatus*): a review, p 485–495. In Science and cultivation of edible and medicinal fungi: Mushroom Science XVII: Proceeding of the 17th Congress of the International Society for Mushroom Science

[66] Nagy A, Manczinger L, Tombácz D, Hatvani L, Gyõrfi J, Antal Z, Kredics L. 2012. Biological control of oyster mushroom green mould disease by antagonistic Bacillus species. Biol Control Fungal Bact Plant Pathog 78:289–293.

[67] Brevundimonas 2B and application thereof. 2019. China Patent CN110684700B

[68] Nazir R, Tazetdinova DI, van Elsas JD. 2014. Burkholderia terrae BS001 migrates proficiently with diverse fungal hosts through soil and provides protection from antifungal agents. Front Microbiol 5:598. doi:10.3389/fmicb.2014.00598 25426111 PMC4227525

[69] Hervé V, Ketter E, Pierrat JC, Gelhaye E, Frey-Klett P. 2016. Impact of Phanerochaete chrysosporium on the functional diversity of bacterial communities associated with decaying wood. Plos One 11:e0147100. doi:10.1371/journal.pone.0147100 26824755 PMC4732817

[70] Bánfi R, Pohner Z, Szabó A, Herczeg G, Kovács GM, Nagy A, Márialigeti K, Vajna B. 2021. Succession and potential role of bacterial communities during Pleurotus ostreatus production. FEMS Microbiol Ecol 97:fiab125. doi:10.1093/femsec/fiab125 34498665 PMC8445668

[71] Warmink JA, Nazir R, van Elsas JD. 2009. Universal and species-specific bacterial “fungiphiles” in the mycospheres of different basidiomycetous fungi. Environ Microbiol 11:300–312. doi:10.1111/j.1462-2920.2008.01767.x 19196267

[72] McBee RM, Lucht M, Mukhitov N, Richardson M, Srinivasan T, Meng D, Chen H, Kaufman A, Reitman M, Munck C, Schaak D, Voigt C, Wang HH. 2022. Engineering living and regenerative fungal-bacterial biocomposite structures. Nat Mater 21:471–478. doi:10.1038/s41563-021-01123-y 34857911

[73] Lawson CE, Harcombe WR, Hatzenpichler R, Lindemann SR, Löffler FE, O’Malley MA, García Martín H, Pfleger BF, Raskin L, Venturelli OS, Weissbrodt DG, Noguera DR, McMahon KD. 2019. Common principles and best practices for engineering microbiomes. Nat Rev Microbiol 17:725–741. doi:10.1038/s41579-019-0255-9 31548653 PMC8323346

