# Supplementary material for: Establishing microbial communities to promote the growth of Pleurotus ostreatus through a top-down approach is hindered by the dominance of antagonistic interactions
Source: Appl Environ Microbiol. 2025 Aug 25;91(9):e00898-25. doi: 10.1128/aem.00898-25 (PMC12442370; doi:10.1128/aem.00898-25)

Figure S1 : *P. ostreatus* growth bag. After transferring substrate into the bag, the opening of the latter was inserted into a plastic collar. The ends of the bags were folded around the collar and secured with an elastic band. The collar was closed using a black threaded polypropylene cap (22 mm, hole 586") with a porous membrane from which the membrane had been removed beforehand. The hole in the center of the cap (initially filled by the membrane) was then covered with a strip of 3M® Micropore medical tape (5 cm x 9.1 m).

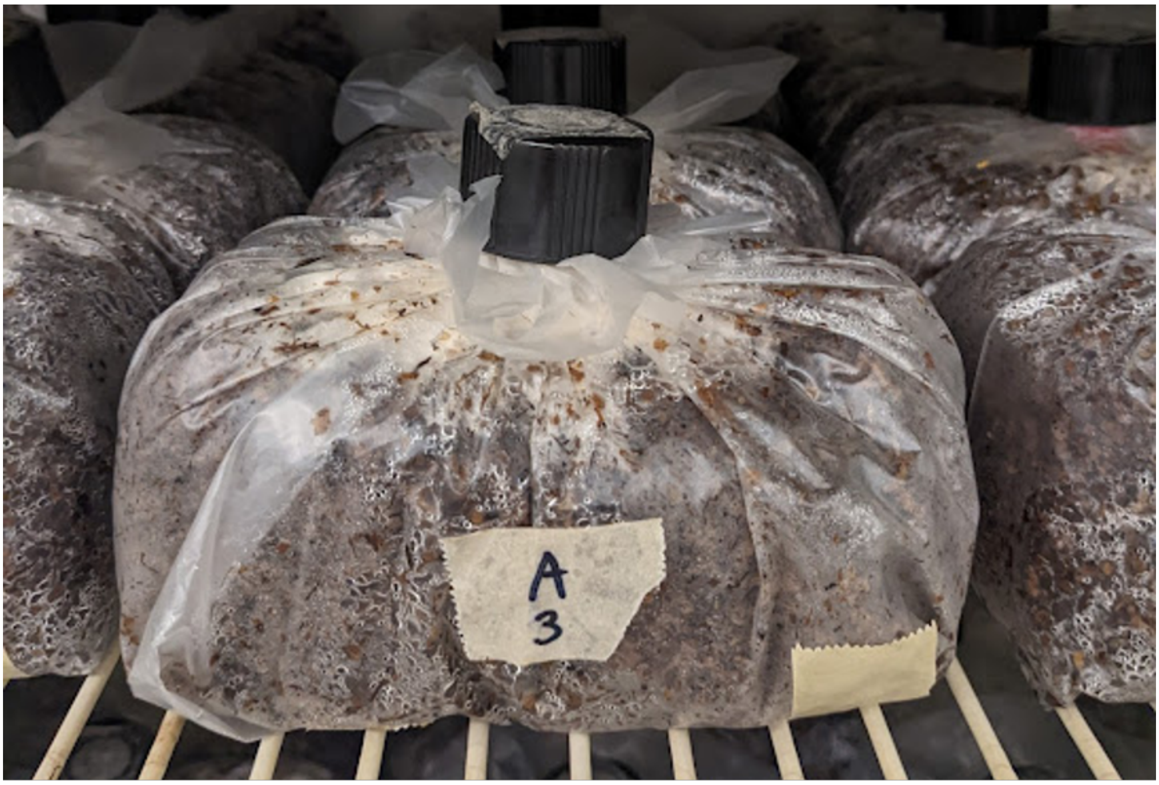

Figure S2 : Confrontation between *P. ostreatus* and *Brevundimonas* sp. MB49 in lignocellulosic substrate-based assays.

A lignocellulosic substrate-based assays, corresponding to Petri dish filled with lignocellulosic substrate, was assessed in which the growth of the fungus and the bacteria were monitored. An overnight *Brevundimonas* sp. MB49 culture in Tryptic Soy Broth (TSB) was adjusted with a final OD_{600} of 2 in sterile PBS 1X. The diluted culture was then washed and the pellet was resuspended to a final volume of 15 ml of PBS 1X. Mycelial plugs of *P. ostreatus* (75 mm diameter) were collected from the edge of growing colony after seven incubation days and transferred into sterilized rye seed for an additional weeks of incubation. The lignocellulosic substrate was prepared following the same proportion as before without the addition of water. The substrate preparation was added to 12 Erlenmeyer flasks and were sterilized twice with an interval of 24h. Prior to inoculation, 350 µl of an ethanol-triton solution was added to the lid of each Petri dish to prevent the formation of condensation. Each Erlenmeyer flask containing the sterilized lignocellulosic substrate was hydrated with either 15 ml of PBS 1X or the bacterial suspension. The moistened substrate was gently stirred using a sterile spatula. The substrate was then carefully transferred into a sterile Petri dish. A total of 12 plates were prepared for the assay including a control with *P. ostreatus* alone (P), a treatment with *Brevundimonas* sp. MB49 alone (B), a treatment with *P. ostreatus* co-inoculated with *Brevundimonas* sp. MB49 (BP) and a treatment without microbial inoculation (Control). For the treatment with *P. ostreatus* alone (P) and the co-inoculation treatment (BP), rye seed colonized by *P. ostreatus* mycelium was placed at the center of the petri dish previously filled with moistened lignocellulosic substrate. All plates were incubated in the dark at 25°C. The growth of *P. ostreatus* mycelium was evaluated by measuring the area of the mycelium (mm^2) with ImageJ after 5 days of incubation. The biomass of *Brevundimonas* sp. MB49 was assessed by Droplet Digital PCR (ddPCR). On Day 0, 3 samples of 0.1 g of lignocellulosic substrate were collected from 3 distinct positions within substrate for each trials. On Day 5, 9 samples of 0.1 g of substrate were collected for the BP treatment, for which 3 samples were collected in the mycelium area (BPM), 3 samples were collected from the edge of the mycelium (BPR) and 3 were collected at the edge of the microcosm without mycelium (BPE). For the other treatments (P, B and C), 3 samples of 0.1 g were collected from 3 distinct positions within substrate (see Supplementary Figure 2). All collected samples were stored at -20°C. Total DNA was extracted from all samples with the DNAeasy® Plant® Pro Kits, (QIAGEN®), following the manufacturer's instructions. Droplet digital PCR was performed on a BioRad QX200 system utilizing the Evagreen assay. The DNA extracted from day 0 and day 5 samples were amplified using the Universal primers 16S_520F

(5' AGCAGCCGCGGTAAT 3') and 16S_799R2 (5' CAGGGTATCTAATCCTGTT 3') to target the V4 region of bacterial 16S rRNA gene (279 pb). Each reaction contained 11.25 µl of Evagreen supermix (Bio-Rad, Hercules, USA), 1.125 µl of each primer, 2.9 µl of milli-Q water and 5 µl of each DNA sample. After the generation of droplet with QX200 droplet Generation Oil for Evagreen (Bio-Rad, Hercules, USA), the ddPCR was performed with a C1000 Touch Thermal Cycler (Bio-Rad, Hercules, USA) with the following cycling program : 5 min at 95°C, 50 cycles of 30s at 95°C, 1 min at 52,1°C and 1 min at 72°C, followed by 5 min at 4°C and 5 min at 90°C (see Supplementary Data). The ramp rate for all the steps was set to 2°C/s. After PCR amplification, the plates were transferred into the QX200 droplet reader (Bio-Rad, Hercules, USA) for fluorescence detection, where positive and negative droplets were distinguished based on manual threshold fluorescence intensity setting. Copy number concentrations were converted to copy per gram of lignocellulosic substrate.

Schematic overview of the sampling strategy of the microcosm confrontation bioassay. The different treatment corresponds to : *P. ostreatus* alone (P), *Brevundimonas* sp. MB49 alone (B), co-inoculation of *P. ostreatus* and *Brevundimonas* sp. MB49 (BP) and a control (C). Samples were collected just after inoculation and after 5 days of incubation. The white circle represent the mycelium of *P. ostreatus*. The different sampling position correspond to : Orange, samples in the mycelium area (BPM); blue, samples from the edge of the mycelium (BPR); yellow, samples at the edge of the microcosm without mycelium (BPE).

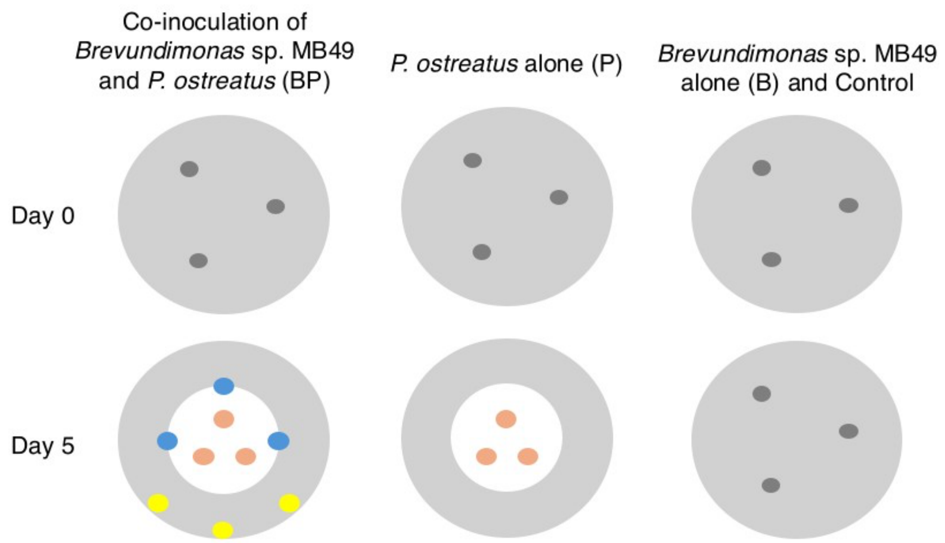

Figure S3. Relative abundance of the abundant fungi phylum (A) and genera (B) across the different treatment in the enrichment cultures and in the lignocellulosic substrate.

A.

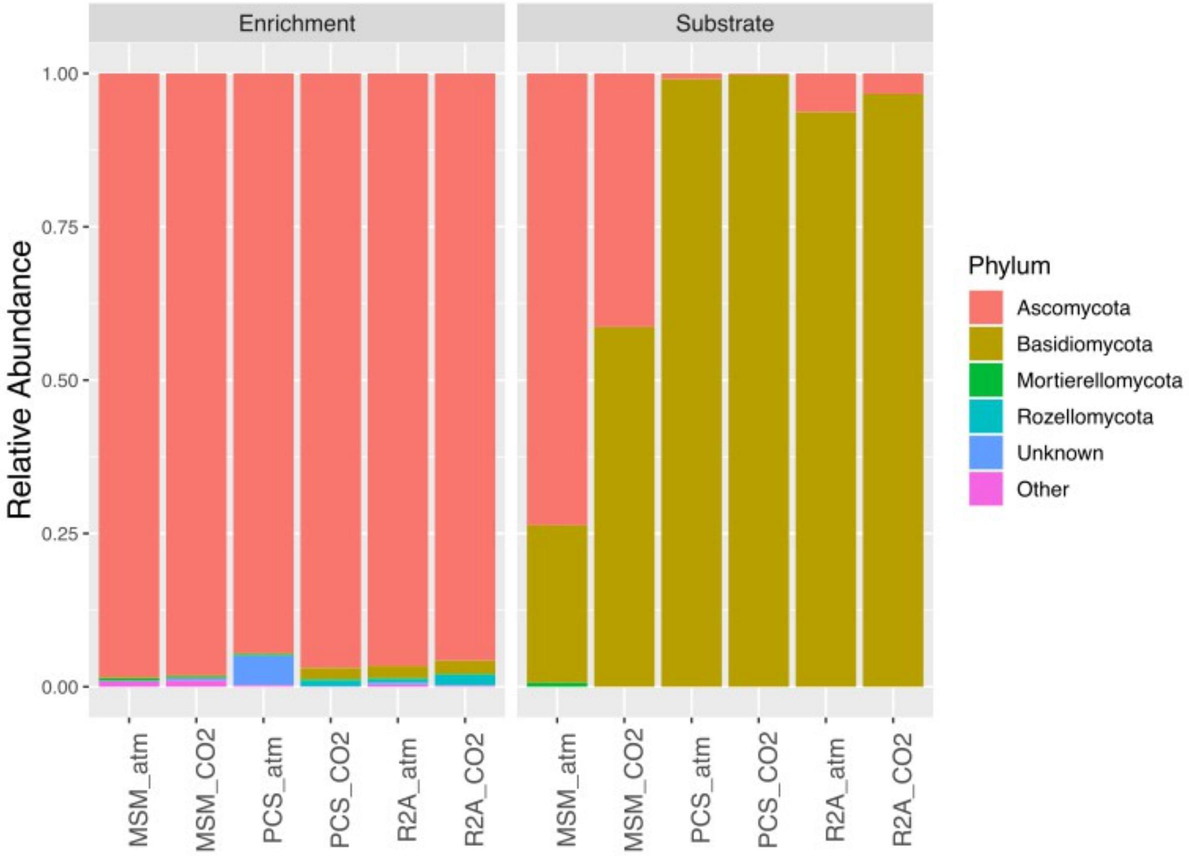

B.

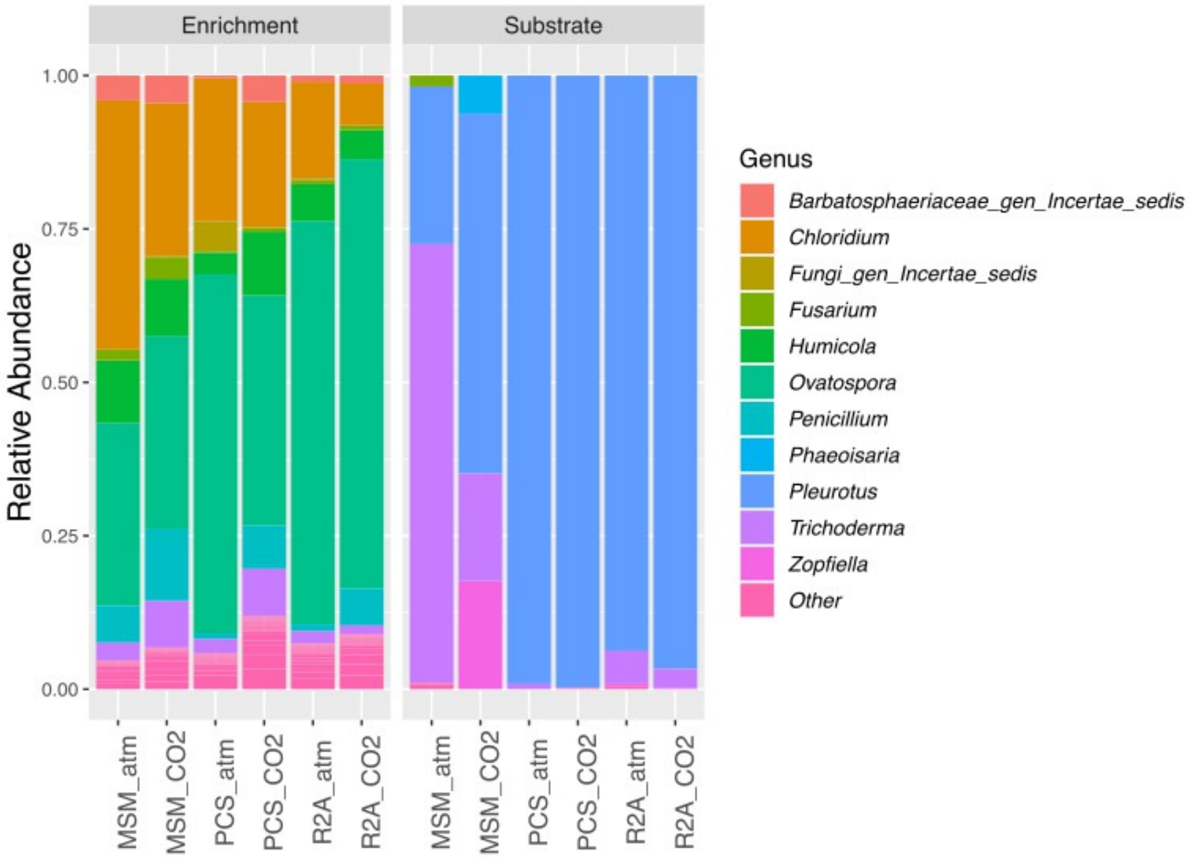
Enrichment
Substrate
Relative Abundance
1.00
0.75
0.50
0.25
0.00
MSM_atm
MSM_CO2
PCS_atm
PCS_CO2
R2A_atm
R2A_CO2
MSM_atm
MSM_CO2
PCS_atm
PCS_CO2
R2A_atm
R2A_CO2
Genus
Barbatosphaeriaceae_gen_Incertae_sedis
Chloridium
Fungi_gen_Incertae_sedis
Fusarium
Humicola
Ovatospora
Penicillium
Phaeoisaria
Pleurotus
Trichoderma
Zopfiella
Other

Figure S4 : Observation of Mycelium Distribution in MSM-G Assay (right) vs. Control Assay (left) after 14 days of incubation. In the control assay, mycelium proliferation encompasses the entire substrate surface. In MSM-G assay, the mycelium exhibits a restricted growth pattern, confined predominantly to the area surrounding the inoculation spot. This replicate exemplifies observations consistent across all test replicates embedded with enrichment.

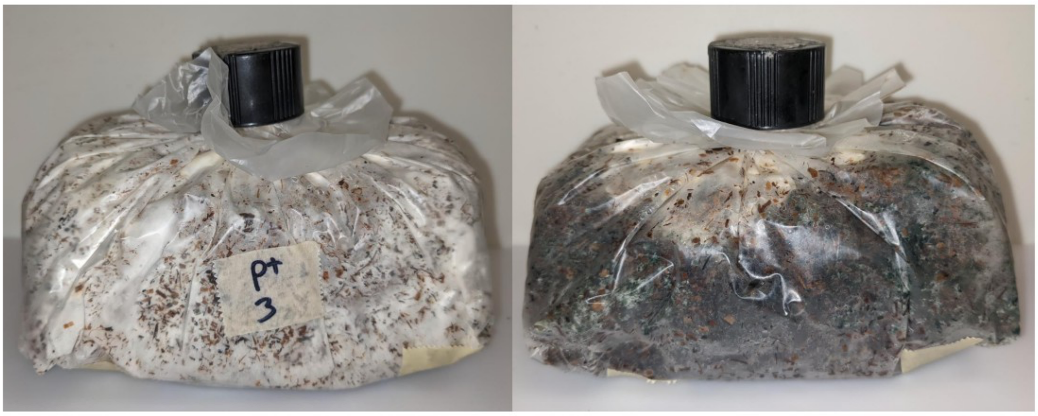

Figure S5. *Brevundimonas* sp. MB49 abundance in the lignocellulosic substrate-based bioassays after inoculation and after 5 days of incubation. The different treatment corresponds to : *P. ostreatus* alone (P), *Brevundimonas* sp. MB49 alone (B), co-inoculation of *P. ostreatus* and *Brevundimonas* sp. MB49 (BP), with sample collected in the senescent mycelium area (BPM), with samples collected from the edge of the mycelium (BPR) and samples collected at the edge of the substrate without mycelium (BPE) and a control (C). Letters differ by Tukey's test ($P < 0.05$).

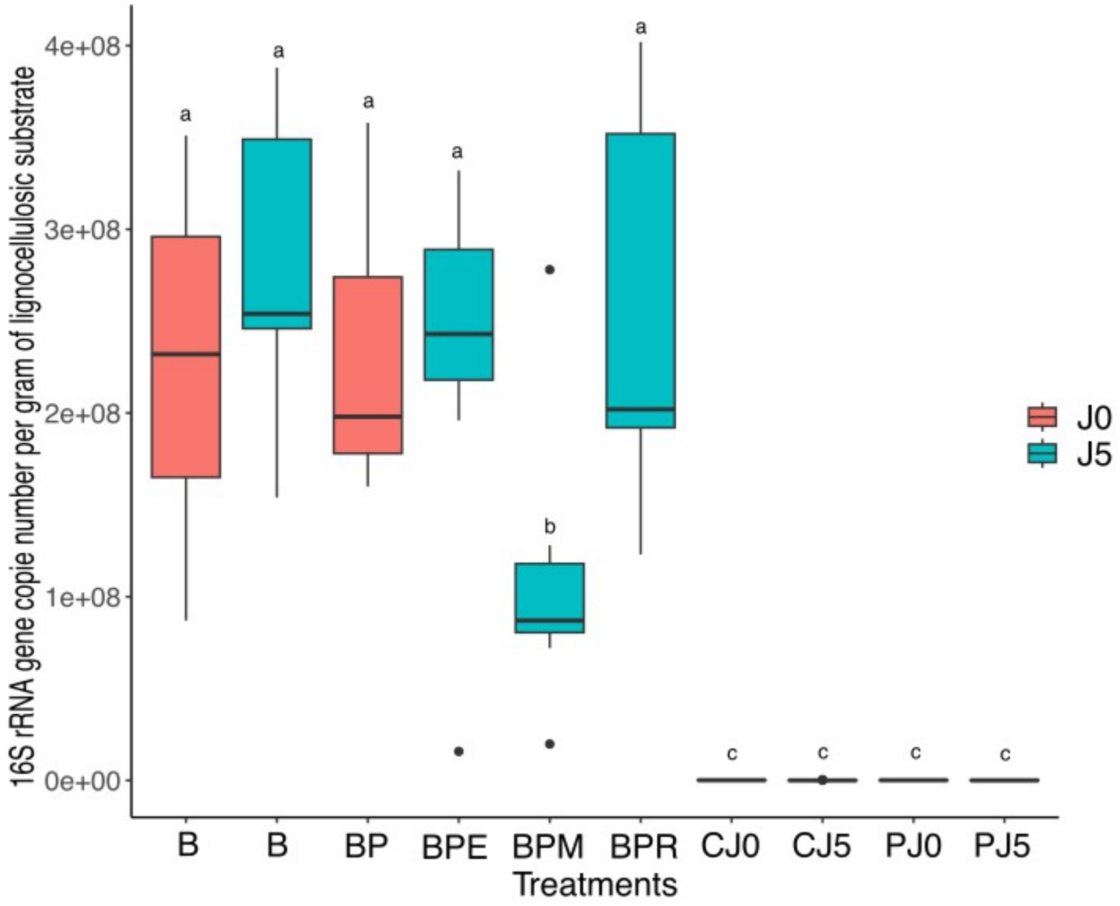

Supplement: Supplemental figures — Figures S1 to S5. [file aem.00898-25-s0001.pdf]
